# Transcriptome Analysis Reveals Cross‐Kingdom Virulence Factors in *Erwinia persicina* Cp2

**DOI:** 10.1111/1758-2229.70322

**Published:** 2026-03-11

**Authors:** Rong Huang, Xiao‐Ni Liu, Tom Hsiang, Zhen‐Fen Zhang

**Affiliations:** ^1^ Pratacultural College, Gansu Agricultural University Lanzhou China; ^2^ Environmental Sciences, University of Guelph Guelph Ontario Canada

**Keywords:** cross‐kingdom, *Erwinia persicina*, grassland agroecosystems, phytopathogen, zoopathogen

## Abstract

*Erwinia persicina* is a well‐documented plant pathogenic bacterium, causing soft rot in various plant hosts. There are rare previous reports that it is associated with animal diseases. In this study, we used an alfalfa root infection model and a mouse model via gavage administration of *E. persicina* strain Cp2. Strain Cp2 was pathogenic on both alfalfa and mice. On alfalfa, Cp2 primarily caused leaf spot and etiolation. Mice exhibited moderate lesions in their liver, stomach and kidney tissues, with inflammation in the liver and kidney after gavage. Furthermore, viable Cp2 cells were successfully reisolated from mouse faeces at different time periods after gavage. Transcriptome analysis revealed that the 34 bacterial differentially expressed genes (DEGs) in Cp2 in response to infecting both alfalfa and mice were predominantly enriched in pathways related to bacterial adhesion, biofilm and motility. *Bap* was identified as a key virulence gene contributing to cross‐kingdom pathogenicity of Cp2. The results provide a solid foundation for the subsequent functional validation of the virulence factors of this cross‐kingdom pathogenic bacterium.

## Graphical abstract

Pathogenic mechanism of *Erwinia persicina* Cp2 across plant and animal kingdoms.

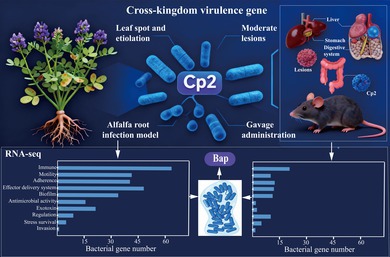

## 1 Introduction

*Erwinia persicina* is a gram‐negative, rod‐shaped, facultative anaerobic, flagellated facultative anaerobic bacterium that produces water‐soluble pink pigments and belongs to the family Enterobacteriaceae (Brenner et al. 2005; Zhang et al. 2022). This bacterium shows a remarkably wide ecological distribution, having been isolated from diverse sources, including fungi (Yan et al. 2018), plants (Orel 2020; Nechwatal and Theil 2019; Gálvez et al. 2015), insects (Kim et al. 2022) and animals and even inanimate objects such as ancient frescoes (Hao et al. 2020) and rocks (Kim et al. 2022). In plants, *E. persicina* acts as an opportunistic pathogen capable of causing tissue necrosis and soft rot diseases on hosts such as *Medicago sativa* (Zhang and Nan 2012), *Phaseolus vulgaris* (González et al. 2005, 2007) and *Pisum sativum* (Wasendorf et al. 2022). It can also cause plant tissue death, with pink lesions observed on the roots of *Petroselinum crispum* (Nechwatal and Theil 2019) and *Allium cepa* (Gálvez et al. 2015). In animal trials, *E. persicina* was found to cause flatulence, eye hyperaemia and lymphatic inflammation in *Ovis aries* , as well as liver necrosis and disseminated intravascular coagulation of kidney tissue in mice (Mohamaden et al. 2019). In addition, clinical human studies have revealed that *E. persicina* can also cause urethritis (O'Hara et al. 1998). Notably, our laboratory isolated the *E. persicina* Cp2 strain from alfalfa seeds and demonstrated that it caused wilting and necrosis in 20 species of legume forage crops, including *M. sativa* and *Glycine max* , via a suspension pre‐soak method (Zhang and Nan 2014). Collectively, these studies suggest that *E. persicina* is a cross‐kingdom pathogen capable of infecting plants and animals.

Evidence suggests that some of the underlying mechanisms of bacterial pathogenesis may be similar in the plant and animal kingdoms. This concept is supported by the existence of cross‐kingdom pathogens such as *Pseudomonas aeruginosa* (Rahme et al. 1995) and *Pantoea agglomerans* (Lee et al. 2010). Moreover, bacterial proteins involved in the export of virulence factors (molecules produced by pathogens that enable them to infect and cause disease in a host) have been shown to be conserved between plant and mammalian pathogens (Büttner and Bonas 2003; Kishitani et al. 2000). A notable example is *P. aeruginosa* strain UCBPP‐PA14, a cross‐kingdom pathogen that causes soft rot in *Arabidopsis thaliana* and systemic infection and death in mice. Critically, mutations in the *toxA*, *picS* and *gacA* attenuate its pathogenicity in both hosts, indicating that these genes encode core virulence factors essential for the full expression of pathogenicity in both plants and animals (Rahme et al. 1995). The *toxA*, *picS* and *gacA* mutants caused disease symptoms in *Arabidopsis*, including soft rot and chlorosis. Notably, and in contrast to the wild‐type UCBPP‐PA14, the mutants failed to induce the characteristic water‐soaking and collapse of leaf tissues. In the mouse model, mice that were burned and infected with either *toxA*, *plcS* or *toxA* mutants exhibited significantly lower mortality (40% with both mutants) as compared to infection with the wild‐type strain (77%) (Rahme et al. 1995). The existence of such ‘dual’ plant–animal pathogens raises intriguing evolutionary questions, particularly regarding whether shared virulence factors mediate pathogenesis on members of both kingdoms (Rahme et al. 1995). Recent advances in transcriptomics have emerged as powerful tools to dissect and understand the dynamic interplay between hosts and pathogens (Westermann et al. 2016; Lum and Cristea 2016).

In this study, we used RNA‐seq technology and bioinformatic methods to investigate the key virulence genes linked to *E. persicina* Cp2 infection. Given that *E. persicina* can enter the bodies of animal hosts through the food chain in grassland agroecosystems, we designed a dual‐model system to evaluate its pathogenic mechanisms: (1) an alfalfa root infection model to simulate plant pathogenesis, and (2) a mouse model to assess mammalian infection. Through these systems, we aimed to delineate conserved virulence strategies underlying cross‐kingdom pathogenicity. Preliminary findings from the alfalfa infection experiments have been published (Yao et al. 2022). Here, we expand upon this work by integrating transcriptomic analyses to elucidate bacterial virulence determinants. Our results establish a solid foundation for the further exploration of key virulence genes and pathways associated with cross‐kingdom infection by *E. persicina* Cp2, providing a theoretical basis for further studies on disease prevention and control.

## 2 Materials and Methods

### 2.1 Bacterial Strain

The *E. persicina* strain Cp2 (NCBI accession: 756944), originally isolated from the internal tissue of alfalfa seeds, was obtained from the *Laboratory of Forage Pathology*, *Pratacultural College*, Gansu Agricultural University (Zhang 2013). For experimental use, the strains were inoculated onto nutrient agar (NA: 3 g beef extract, 10 g peptone, 5 g NaCl, 18 g agar and 1000 mL H_2_O) plates and incubated at 28°C overnight. A single colony was picked from an NA plate, inoculated into liquid nutrient broth (NB) and grown at 30°C for 36 h, followed by centrifugation at 12,000 × *g* for 10 min at 30°C. The bacterial cells were then dissolved in sterile water to the desired concentration as estimated using optical density for subsequent experiments.

### 2.2 Plant Model

The alfalfa cultivar ‘Juneng 995’ was selected for root infection assays, as previously established by Yao et al. (2022). The experiment was conducted at the climatic chamber for the *Pratacultural College*, Gansu Agricultural University. Climatic chamber temperature throughout the plant growth period ranged from 23°C to 25°C by day and from 20°C to 23°C by night, and the relative humidity was 70%–78%. The climate chamber light intensity was 19,000 lx. Plant materials showed symptoms 21 days after being inoculated with 10^9^ CFU/mL Cp2, showing leaf spots and yellowing. For transcriptomic analysis, bacterial suspensions and alfalfa root infection model followed Yao et al. (2022). After 21 days of growth, symptomatic and healthy leaves were collected, flash‐frozen in liquid nitrogen and stored at −80°C until RNA extraction.

### 2.3 Animal Model and Experimental Design

Specific pathogen‐free (SPF) KM mice weighing 18–22 g were used for the experiment, half male and half female and were supplied by *Lanzhou Veterinary Research Institute*, Chinese Academy of Agricultural Sciences. Mice were housed in the SPF Grade Trial Animal Center of Lanzhou University in a temperature‐controlled (22°C ± 1°C) and humidity‐controlled (50% ± 5%) environment.

After 48 h of acclimatisation, mice were randomly divided into two experimental groups, with 8 mice in each group, named the CK group (NB solution) and the Cp2 group (10^9^ CFU/mL Cp2 bacterial suspension). Each group was comprised of 4 male and 4 female mice, housed at a density of 4 animals per cage. Experiments were initiated after the acclimatisation period, and the prepared bacterial suspension was transferred with a sterile gavage needle. The volume of gavage for each mouse was 0.4 mL. Control mice were treated with an equivalent volume of sterile NB. The mice were subjected to fasting for 4 h and water deprivation for 4 h prior to intragastric administration, and then they were given food and drinking water 4 h after gavage. Intragastric administration was performed twice a day for three consecutive days. The animal experiments were approved by the animal ethics committee of Gansu Agricultural University (Licence: SCXK (Gan) 2010‐0002).

### 2.4 Preparation and Observation of Paraffin Sections

At 24, 48, 72 and 96 h after the last gavage, the mice were dissected. A small portion of tissue samples (10 g) was immediately frozen in liquid nitrogen and stored at −80°C until transcriptome analysis. The remaining heart, liver, lung, spleen, stomach and kidney tissues were fixed with 4% paraformaldehyde for more than 24 h. Then, the fixed tissues were sent to Wuhan Saiweier Company (Sevier Biological Technology Co. Ltd., Wuhan, China) for the preparation of paraffin sections. All sections were scanned and analysed with the TissueFAXS imaging system (Tissue Gnostics, Vienna, Austria).

### 2.5 RNA Extraction and Illumina Sequencing

#### 2.5.1 Sample Preparation

Based on observations of the pathological sections of organ tissues in mice, stomach tissues exhibited the most severe lesions, including gastric epithelial cell sloughing and abnormal gland dropout structure. Thus, samples for transcriptome analysis were selected from pathological stomach tissues of mice (Cp2‐A(Mice)). As well, Cp2 bacterial cells (Cp2), healthy alfalfa leaves (P(Alfalfa)), healthy mouse stomach tissues (A(Mice)) and healthy and diseased junctions from alfalfa leaves (Cp2‐P(Alfalfa)) were chosen. For RNA extraction from plants, diseased leaves were cut into small pieces (0.5 × 0.5 cm^2^) by using a sterile scalpel at the interface between healthy and diseased leaves. Then, these samples were placed in 5 mL grinding tubes (Biosharp Co. Ltd., Shanghai, China), crushed and ground, with high‐throughput tissue grinder equipment (Scientz‐48L; Ningbo Scientz Biotechnology Co. Ltd). Healthy leaves were processed using the same method.

According to the results of the imaging system, the stomach tissue of mice showed the most serious pathogenic symptoms at 96 h after gavage. Here, we selected stomach tissue samples at these time points for dual RNA‐seq analysis. The stored stomach tissue samples were taken from the −80°C refrigerator, and the tissues were homogenised, and total RNA was extracted for subsequent analysis. Bacterial RNA and alfalfa total RNA were isolated using the YEASEN Bacterial RNA Extraction Kit (19301ES50) and the Plant RNA Extraction Kit (Tiangen Biology Company, DP432, Beijing, China), respectively, in accordance with the manufacturers' instructions. The TRIzol reagent (Tiangen Biology Company) was used to extract the total RNA from the stomach tissue of mice. Three biological replicates were set for each sample. RNA concentration and purity were evaluated using an Agilent 2100 bioanalyzer (Agilent Technologies, CA, USA), and RNA integrity was determined via 1% agarose gel electrophoresis. After RNA extraction, cDNA library construction (NEBNext Ultra Directional RNA Library Prep Kit: NEB, USA, Catalogue #: E7420) and transcriptome sequencing were conducted by Novogene Co. Ltd. (Beijing, China). Briefly, Ribosomal RNA was depleted from total RNA using the Ribo‐off rRNA Depletion Kit (Vazyme Biotech, China). The purified RNA was fragmented and reverse‐transcribed into double‐stranded cDNA. The cDNA fragments were then end‐repaired, adenylated and ligated with Illumina adapters. After PCR amplification, the final libraries were quantified and quality‐controlled on an Agilent 2100 Bioanalyzer. Qualified libraries were sequenced on an Illumina NovaSeq 6000 platform with a PE150 strategy.

#### 2.5.2 Screening of Differentially Expressed Gene (DEG)

RNA sequencing libraries were subjected to PE150 sequencing on a NovaSeq6000. Raw sequencing reads were first assessed for quality using FastQC (v0.11.9). Subsequently, quality control and adapter trimming were performed with fastp (v0.23.2) using the following parameters: a sliding window of 4 bp with a required minimum average quality score of Q20, and the removal of reads shorter than 36 bp after processing. Raw sequencing reads were subjected to quality control and preprocessing to obtain high‐quality clean reads. These reads were mapped to the *E. persicina* , alfalfa and mice reference genomes by using the Subread software package (https://subread.sourceforge.net/). The feature counts function within Subread was used to tally the number of clean reads covered by each gene aligned to the reference genome, ranging from the initial to the terminal regions. Based on the location information of gene alignment in the respective reference genome, the number of reads covered by each gene from start to end was calculated using the feature counts tool, and non‐conforming clean reads were filtered out. The quantification of gene expression was performed using HTSeq version 0.9.1 (https://htseq.readthedocs.io/en/release_0.9.1/), and the FPKM (fragments per kilobase of exon model per million mapped fragments) method was used to eliminate the influence of different gene lengths and sequencing levels on the calculation of gene expression.

The DEGs analysis was performed using the DESeq2 package (version 4.2) from Bioconductor. Briefly, the DESeq2 model incorporated our sample grouping to estimate size factors for normalisation, gene‐wise dispersions and fit a negative binomial generalised linear model. Significant DEGs were then identified based on a threshold of log2 fold change (FC) ≥ 1 and corrected *p* < 0.05. All bacterial DEGs were subjected to Gene Ontology (GO; https://geneontology.org/) and Kyoto Encyclopedia of Genes and Genomes (KEGG; https://www.genome.jp/kegg/) enrichment analyses using GOseq and KOBAS software, respectively. Additionally, virulence factor analysis and pathogenic bacteria–host interaction analysis were performed for all bacterial DEGs. Another strain of plant and animal cross‐kingdom pathogen was selected, *Pantoea alfalfa* sp. nov. CQ10 (CQ10) as a control strain for screening cross‐kingdom virulence factors and pathogen–host interaction genes (data were derived from the transcriptome data of CQ10 with alfalfa and mice produced in another study) (Su et al. 2024). The infection concentration (10^9^ CFU/mL) and inoculation method (alfalfa root infection model and mouse model via gavage) for CQ10 were kept consistent with those for Cp2. Following infection, samples of alfalfa (CQ10‐P(Alfalfa)) and mouse stomach tissue (CQ10‐A(Mice)) were collected at the same time points for subsequent analysis.

### 2.6 RT‐qPCR Validations

To verify the reliability of transcriptome sequencing and the expression of DEGs in the Cp2 strain, we selected eight genes involved in a variety of processes for expression level analysis via RT‐qPCR. The housekeeping gene, RNase P (*rnpB*), was used as the reference. Total RNA from the samples was extracted using a TRIzol Kit (Trademark, TM; Invitrogen, A2010A0402) and reverse transcribed using a FastKing RT Kit (KR116, Tiangen Co. Ltd., Beijing) according to the manufacturer's protocols. All primers used for RT‐qPCR analyses were designed using Primer Premier 5 software and are listed in Table S1. RT‐qPCR experiments were performed using a LightCycler 96 system (Roche, USA), and the reaction system was set up using the SuperReal PreMix Plus (SYBR Green) Kit (Tiangen Co. Ltd.) with a 20 μL reaction. The 2^−ΔΔCt^ method calculation was used for gene expression analysis via normalisation to SaUBC9 (Livak and Schmittgen 2001), and the results were expressed as mean ± standard deviation (SD).

## 3 Results

### 3.1 Effect of Cp2 on Pathological Changes in Mice

**FIGURE 1:**
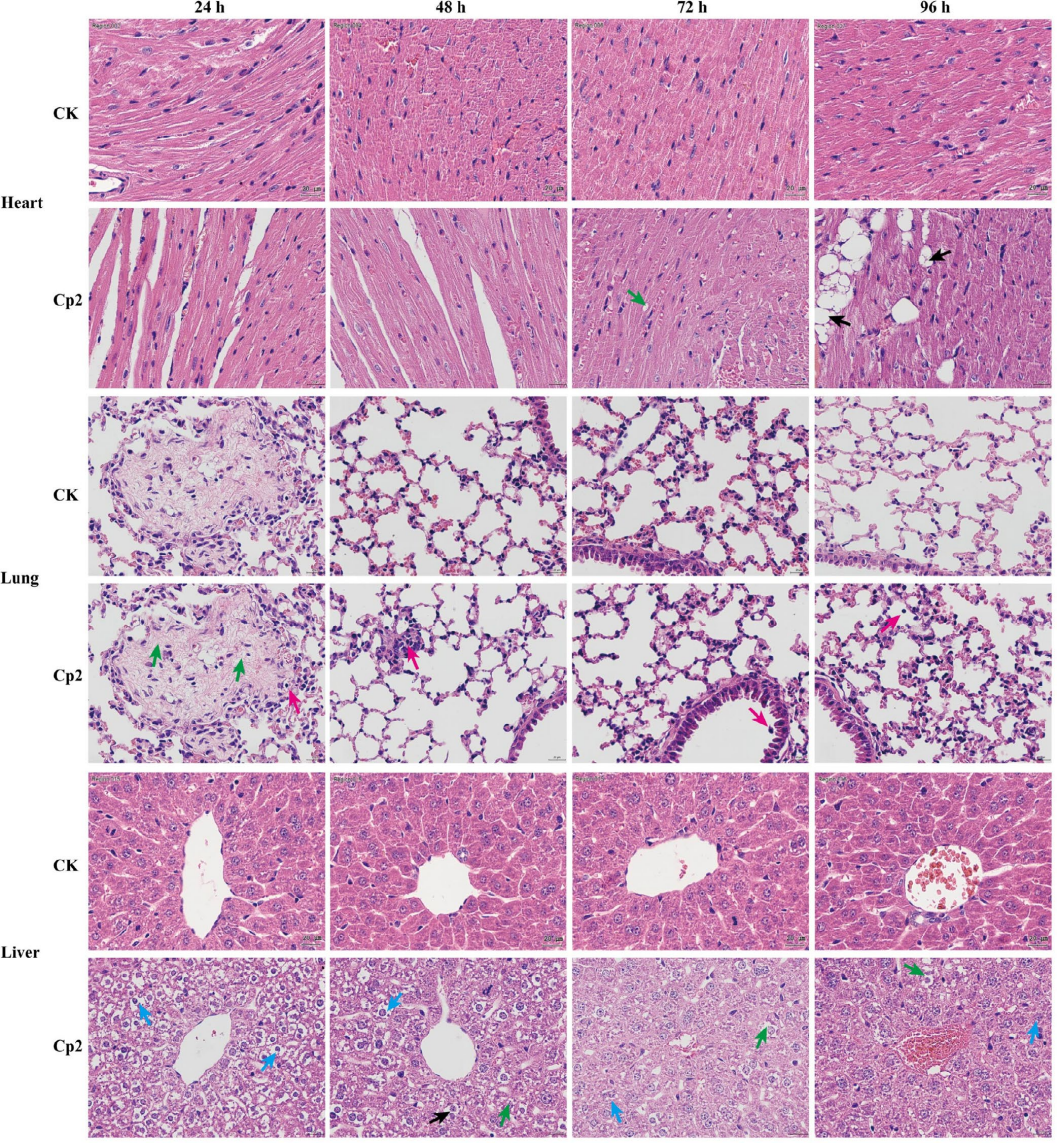
Pathological changes in heart, liver and lung tissues of mice after inoculation. Heart: Green arrows point to cardiomyocytes with mild steatosis and cytoplasmic vacuolation; black arrows indicate adipocyte infiltration between myocardial fibres. Lung: Green arrows point to extensive fibrous tissue hyperplasia; the red arrows indicate minor inflammatory cell infiltration. Liver: Blue arrows point to extensive hepatocellular swelling with cytoplasmic vacuolation; green arrows indicate minor lipid vacuoles; black arrows indicate individual necrotic hepatocytes with karyorrhexis.

**FIGURE 2:**
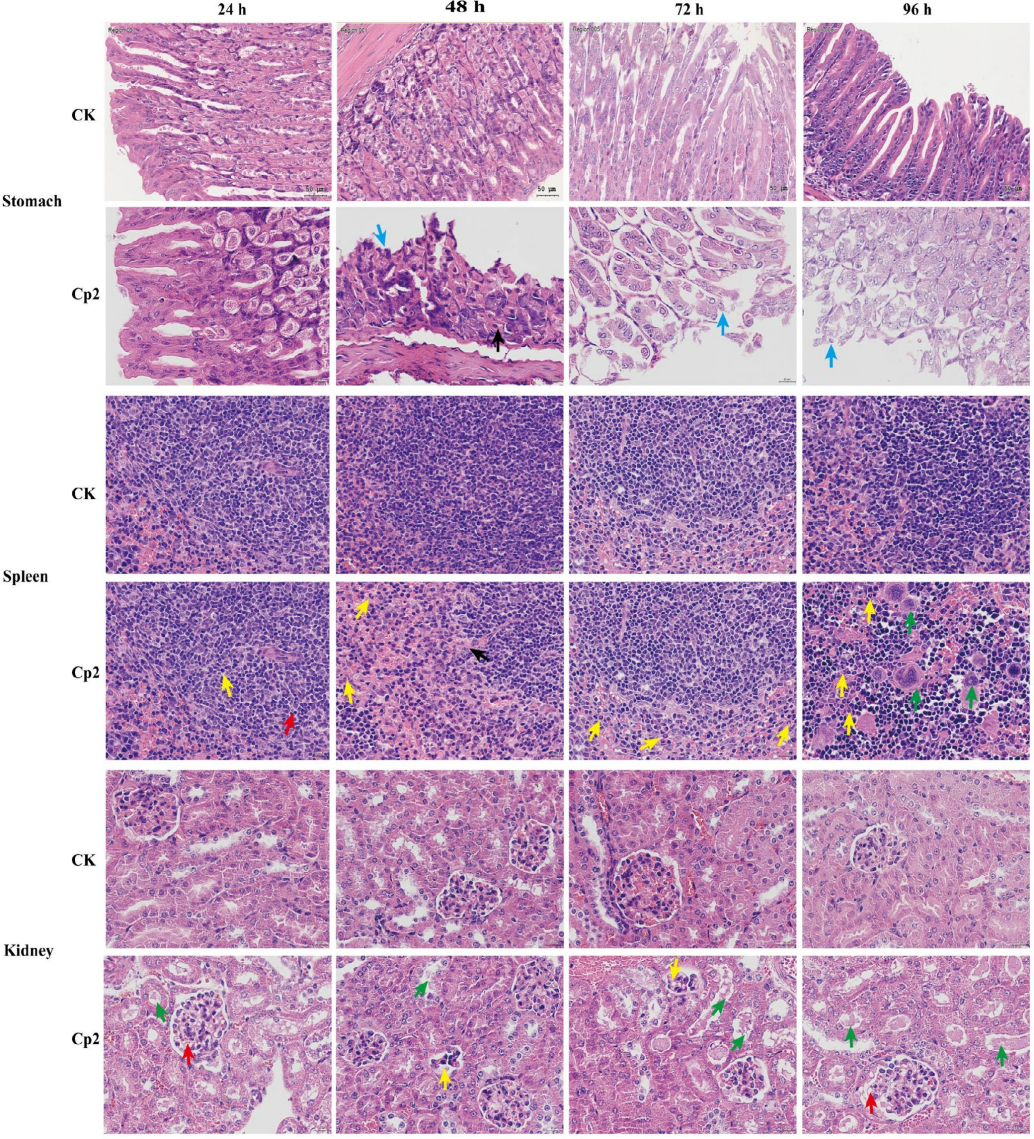
Pathological changes in stomach, spleen and kidney tissues of mice after inoculation. Stomach: Black arrows point to detachment of gastric epithelial cells and exposed lamina propria with disorganised glandular structure; blue arrows indicate minor cell necrosis with karyorrhexis in the lamina propria. Spleen: Red arrows point to focal cell necrosis in the white pulp; yellow arrows indicate minor neutrophil infiltration; black arrows indicate blurred red‐white pulp boundaries. Kidney: Red arrows indicate dilated glomerular capillaries; green arrows point to partial brush border injury; yellow and black arrows indicate lobulated and atrophic glomeruli with reduced mesangial cells.

The heart morphology and function of mice in the Cp2 group appeared normal at 24 and 48 h after gavage (Figure 1). But at 72 h, compared to controls, mild cardiac steatosis was observed, with the cytoplasm of some cardiomyocytes exhibiting vacuolation (green arrows in Figure 1). The cardiac tissue structure exhibited moderate abnormalities, primarily evidenced by the infiltration of adipocytes into the interstices of myocardial fibres 96 h after gavage (black arrows in Figure 1). In the lungs, the alveolar structure was clear, and the alveolar epithelial cells showed no necrosis in the Cp2 group 24 h after gavage (Figure 1). However, extensive fibrous tissue hyperplasia (green arrows in Figure 1) was accompanied by inflammatory cell infiltration (red arrow). Throughout the gavage period, the Cp2 group had persistent and intense inflammatory infiltrates (red arrows in Figure 1). Notably, after 72 h, some sloughed alveolar epithelial cells were present in the alveolar lumen (Figure 1). The hepatic tissue structure of mice exhibited moderate abnormalities, evidenced by diffuse cloudy hepatocellular swelling and vesiculated cytoplasm 24 h after gavage (blue arrows in Figure 1). Symptoms after 48 h showed a similar phenomenon to those observed at 24 h, and a few fat vacuoles were identified (green arrows in Figure 1), in addition to individual hepatocellular necrosis and karyorrhexis in the necrotic hepatocytes (black arrows in Figure 1). After 72 and 96 h, symptoms were alleviated, manifesting as partial hepatocyte oedema, vacuolated cytoplasm, mild fatty degeneration of some hepatocytes and a small number of intracellular fat vacuoles (Figure 1). In the stomach, tissue structure remained normal at 24 h. However, 48 h after gavage, gastric epithelial cells were distinctly exfoliated, with exposed lamina propria that exhibited disorganisation in their glandular structure (black arrows in Figure 2). After 72 h, the symptoms showed some alleviation, characterised by epithelial cell erosion and shedding. After 96 h, there were a few instances of cell necrosis and nuclear fragmentation in the lamina propria of the gastric tissue (blue arrows in Figure 2). In mice spleens 24 h after intragastric administration, there was some cell necrosis (red arrows in Figure 2) in the white pulp, and a small amount of neutrophil infiltration (yellow arrows in Figure 2).

After 48 and 72 h, the spleen tissue structure exhibited moderate abnormalities, indicated by a blurry boundary between the red and white pulp (black arrows in Figure 2), with significant infiltration of neutrophils in the tissue. By 96 h, there was an increase in multinucleated macrophages. In the kidneys, the alterations found 24 h after infection were dilation of the glomerular capillaries (red arrows in Figure 2) and partial brush border injury (green arrows in Figure 2). Cp2‐group mice exhibited lobulation and atrophy of the glomeruli, with a marked decrease in mesangial cells after 48 h (yellow arrows in Figure 2). Symptoms at the 72 h mark were similar to those observed at the 48 h mark, and a small volume of protein mucus was observed in a few glomerular lumina (black arrows in Figure 2). After 96 h, the renal tissue structure remained moderately abnormal (Figure 2). In summary, the mouse model was established through the inoculation of a Cp2 suspension, which revealed that Cp2 can enter the body via feed intake and induce notable lesions in the stomach, liver, kidney and spleen tissues of animals (Figures 1 and 2).

### 3.2 RNA Sequencing Data and Defining DEGs by RNA‐Seq

**FIGURE 3:**
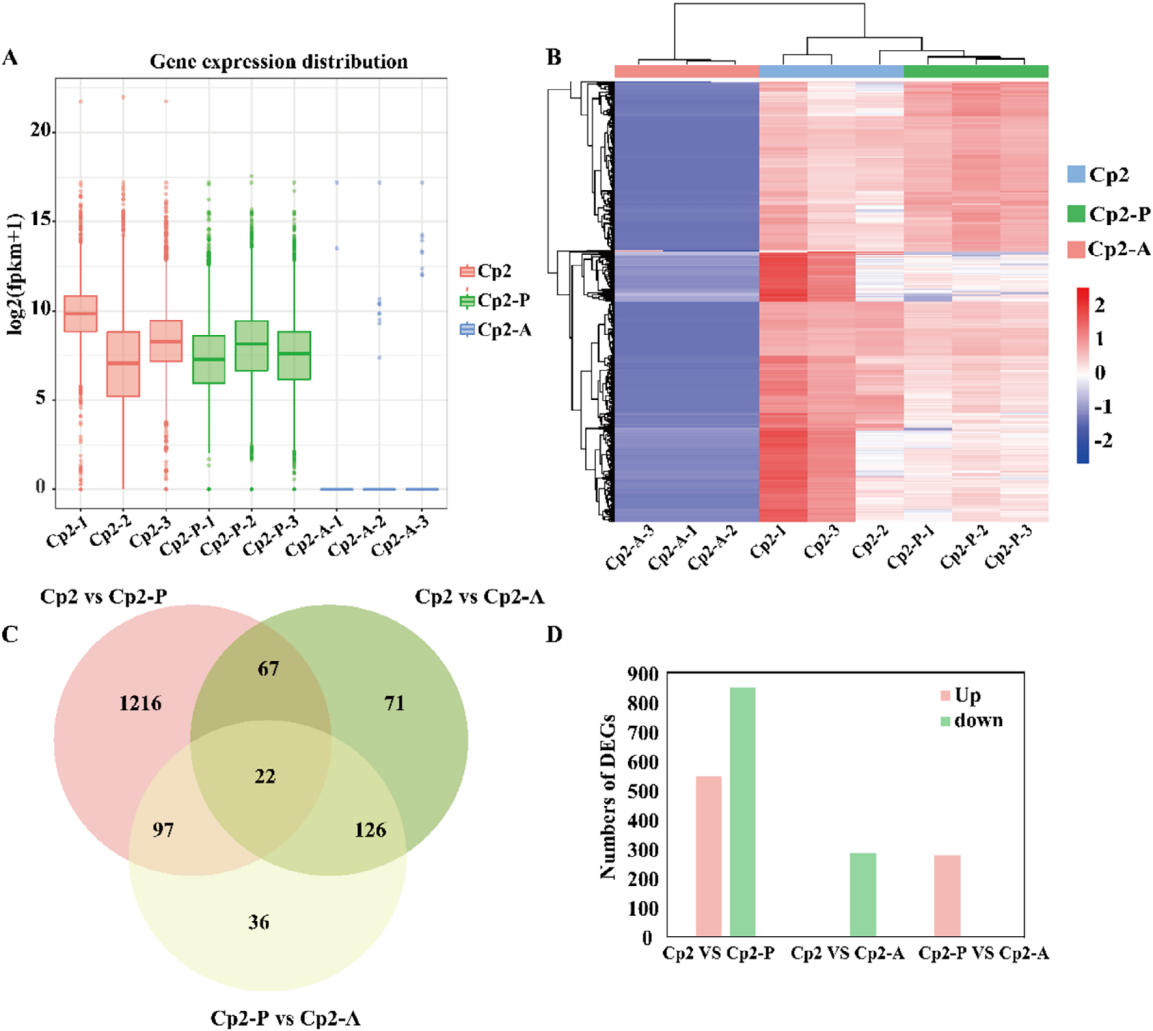
Gene expression distribution and expression analysis of DEGs in three groups. (A) Gene expression distribution in transcriptome. (B) Heatmap and hierarchical clustering of DEGs in three groups. (C) Venn analysis of total DEGs. (D) Number of upregulated and downregulated DEGs. Cp2: Cp2 bacterial cells. Cp2‐A: Animal samples inoculated with Cp2, the same as below; Cp2‐P: Plant samples inoculated with Cp2; DEGs: Differentially expressed genes; ‘up’ and ‘down’ separately represent up/downregulated expression of genes, the same as below.

Fifteen samples, including biological replicates, were subjected to transcriptome sequencing, and 1,378,556,364 clean reads were obtained after filtering out low‐quality reads, resulting in 206.5 Gb of sequence data. The Q20 and Q30 of each sample were higher than 98% and 92%, respectively. The error rates of three libraries (Cp2, Cp2‐P and Cp2‐A) were 0.03%, and those for two libraries (P and A) were 0.07% (Table 1). The log values for the expression (fragments per kilobase of exon model per million mapped fragments) levels of the three groups (Table 1) were between 0 and 12 for each million mapped reads (Figure 3A). After alignment of the clean reads to the Cp2 bacterial library, the average content of the pathogen in infected alfalfa leaves was 3.93%, while that in infected mice was 0.07%. Additionally, the results showed that the total mapping rate in the P and A groups was 0. This result supported matches with *E. persicina* Cp2 genes in Cp2‐P and Cp2‐A groups as really being Cp2 genes and not host genes (Table 2). Hierarchical clustering analysis revealed distinct patterns among the nine samples, with replicates from each treatment clustering together (Figure 3B). The study aimed to determine which bacterial genes were upregulated or downregulated in both mice and alfalfa; thus, we selected a subset of genes commonly expressed in the Cp2‐P and Cp2‐A groups for analysis. A Venn diagram analysis for co‐expressed bacterial genes revealed that the three groups shared 22 DEGs, while there were 89 DEGs between the Cp2/Cp2‐P and Cp2/Cp2‐A groups (Figure 3C). Furthermore, differential expression analysis identified a total of 1969 DEGs. In the Cp2/Cp2‐P group, 1402 bacterial DEGs were found, of which 548 were upregulated, and 854 were downregulated. In the Cp2/Cp2‐A group, there were 286 bacterial DEGs, all of which were upregulated. In the Cp2‐P/Cp2‐A group, there were 281 bacterial DEGs, all of which were upregulated (Figure 3D).

**TABLE 1:** Quality assessment of the RNA‐seq data.

Sample	Raw reads	Clean reads	Error rates (%)	Q20 (%)	Q30 (%)
Cp2‐1	91,222,452	90,609,058	0.03	97.92	93.98
Cp2‐2	86,731,704	85,887,368	0.03	98.03	94.11
Cp2‐3	97,854,972	96,839,262	0.03	98.01	94.05
P‐1	91,537,588	90,141,404	0.07	98.44	95.62
P‐2	97,955,364	96,243,724	0.07	98.27	95.21
P‐3	104,857,300	103,307,640	0.07	98.50	95.80
A‐1	93,883,006	91,992,376	0.07	98.09	94.81
A‐2	104,045,076	101,964,904	0.07	98.12	94.90
A‐3	101,336,564	99,460,776	0.07	98.18	95.07
Cp2‐P‐1	91,843,990	88,688,840	0.03	97.4	92.64
Cp2‐P‐2	86,120,122	83,919,280	0.03	97.17	92.15
Cp2‐P‐3	93,043,318	87,658,908	0.03	97.34	92.5
Cp2‐A‐1	91,762,002	90,077,852	0.03	97.47	92.74
Cp2‐A‐2	82,019,644	80,461,006	0.03	97.5	92.78
Cp2‐A‐3	93,155,532	91,303,966	0.03	97.41	92.9

*Note:* Cp2‐1, Cp2‐2 and Cp2‐3 indicate Cp2 bacterial cells. P‐1, P‐2 and P‐3 indicate the plant group (Alfalfa). A‐1, A‐2 and A‐3 indicate the animal group (Mice). Cp2‐P‐1, Cp2‐P‐2 and Cp2‐P‐3 represent alfalfa infected by the Cp2 strain. Cp2‐A‐1, Cp2‐A‐2 and Cp2‐A‐3 represent mice infected by the Cp2 strain. Q20 and Q30 represent the percentage of the number of bases for which Phred values are greater than 20 or 30 on the total bases, respectively. Error rates represent the percentage of fuzzy bases. The same as below.

**TABLE 2:** The results of the comparison of reads to the reference genome.

Sample	Useful reads	Total mapped reads	Total mapping rates
Cp2‐1	118,360,968	89,489,963	98.8%
Cp2‐2	84,857,606	84,769,054	98.7%
Cp2‐3	91,814,554	95,691,817	98.8%
P‐1	90,141,404	83	0
P‐2	96,243,724	134	0
P‐3	103,307,640	99	0
A‐1	91,992,376	236	0
A‐2	101,964,904	181	0
A‐3	99,460,776	327	0
Cp2‐P‐1	90,153,398	1,661,911	1.9%
Cp2‐P‐2	85,886,728	3,333,425	4.0%
Cp2‐P‐3	107,309,752	5,224,427	4.0%
Cp2‐A‐1	96,568,966	55,591	0.1%
Cp2‐A‐2	81,052,360	24,692	0
Cp2‐A‐3	91,712,820	96,275	0.1%

*Note:* Total mapping rates represent the content of gene expression of strain Cp2 in alfalfa and mice.

To validate the RNA‐seq results, eight genes with significantly different expression levels were selected (*p* < 0.05). These were then investigated in this study as for transcript levels using RT‐qPCR. The RT‐qPCR results matched those of the RNA‐seq data, validating the RNA‐seq data (Figure S1).

### 3.3 GO Functional Classification and KEGG Pathway Enrichment Analysis

**FIGURE 4:**
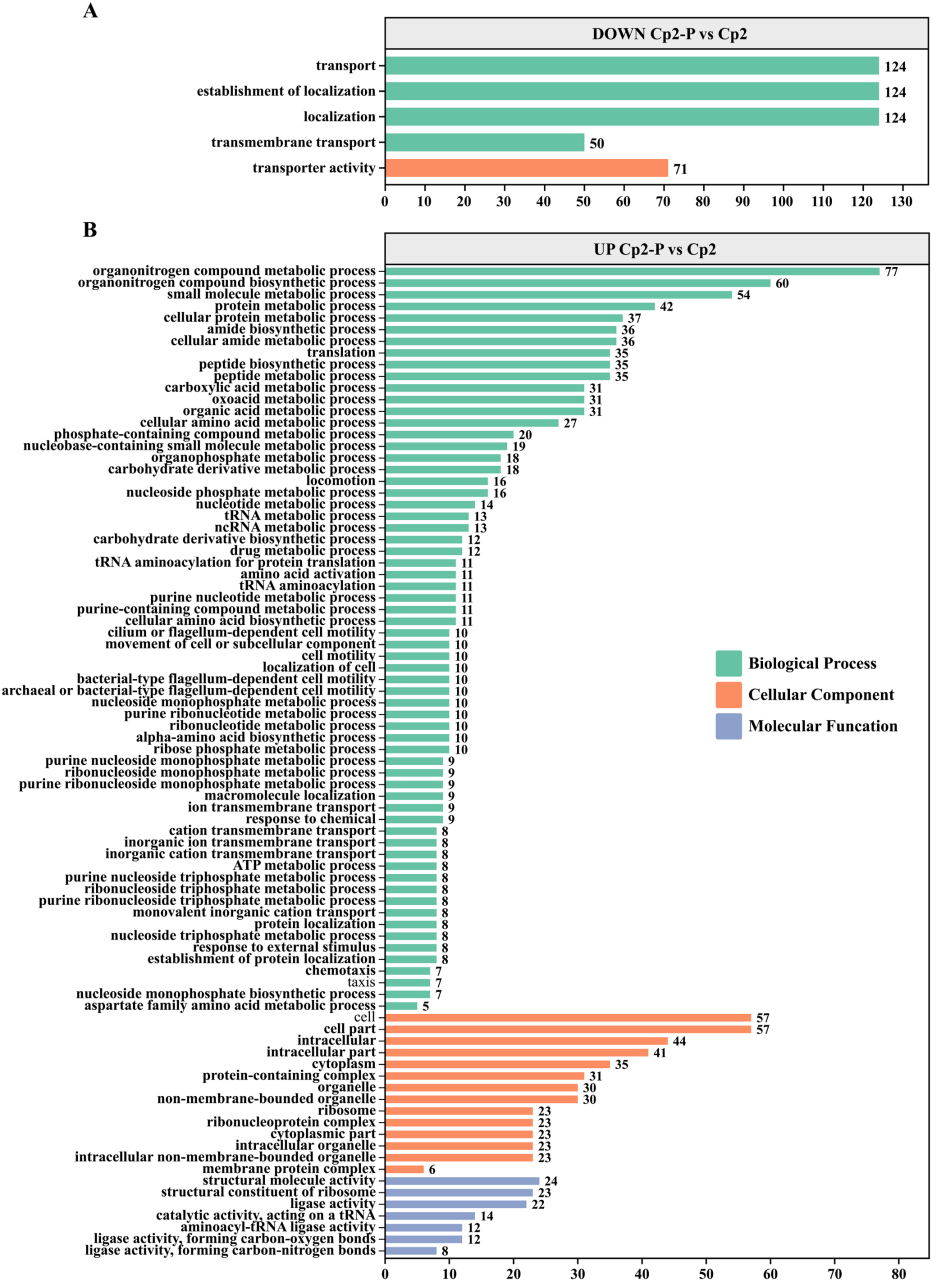
The GO‐annotated entries containing the DEGs. (A) The GO annotated entries containing downregulated DEGs in the Cp2‐P/Cp2 group. (B) The GO annotated entries containing upregulated DEGs in the Cp2‐P/Cp2 group.

**FIGURE 5:**
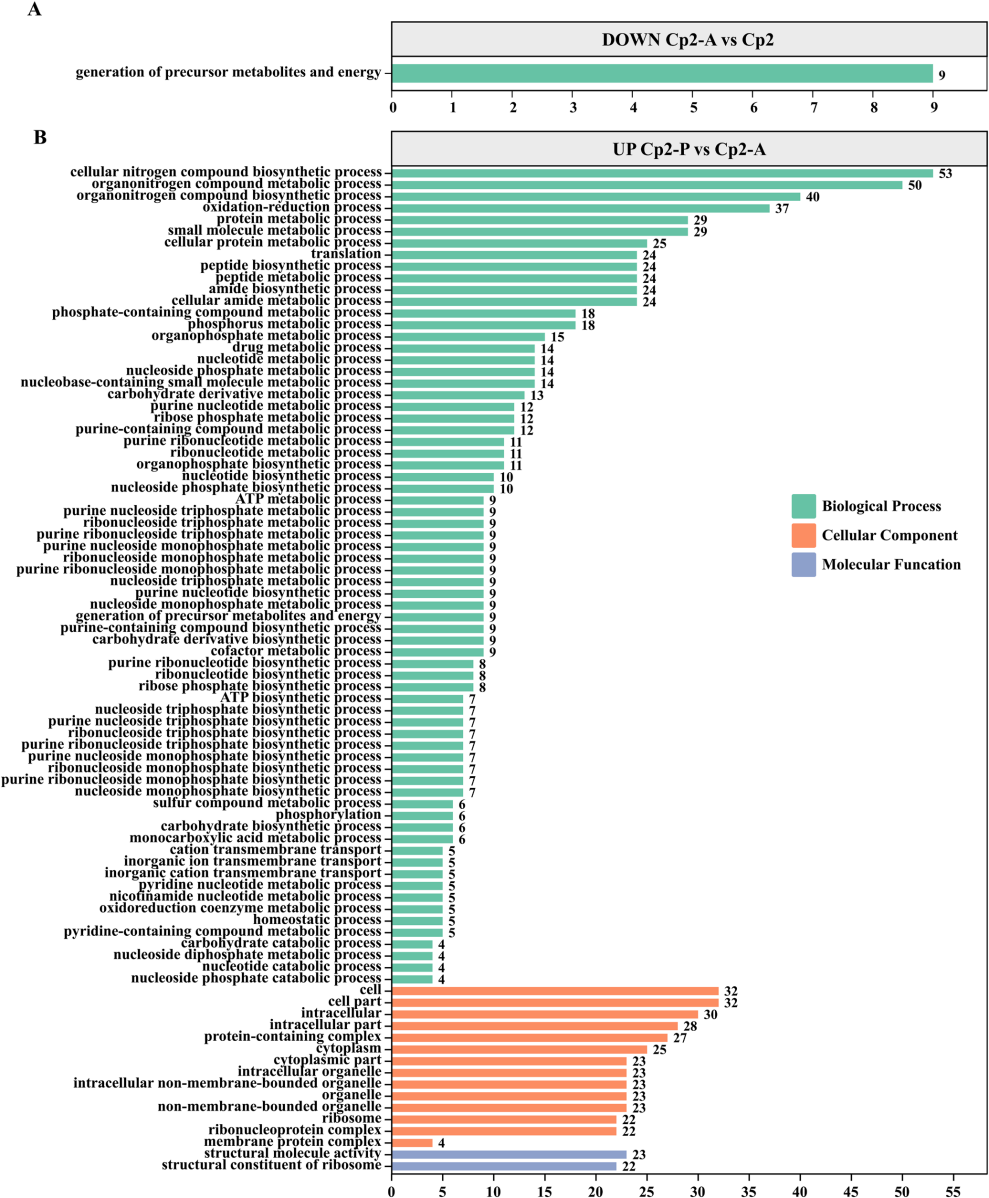
The GO‐annotated entries containing the DEGs. (A) The GO annotated entries containing downregulated DEGs in the Cp2‐A/Cp2 group. (B) The GO annotated entries containing upregulated DEGs in the Cp2‐A/Cp2‐P group.

To elucidate the molecular mechanisms underlying the cross‐kingdom pathogenicity of Cp2, we compared the transcriptomes among the three groups and analysed the coherence level‐2 GO functional annotations (Figure 4 and Figure 5). Overall, GO terms were processed and categorised under three main categories: cellular component, molecular function and biological process. In the Cp2‐P/Cp2 group, the upregulated bacterial DEGs were significantly enriched in a variety of biological processes, including ‘organonitrogen compound metabolic process’, ‘organonitrogen compound biosynthetic process’ and ‘small molecule metabolic process’ (Figure 4B). In the molecular function category, the most enriched terms were ‘cell’, ‘cell part’, ‘intracellular’ and ‘intracellular part’, while the least enriched term was ‘membrane protein complex’. In contrast, the downregulated bacterial DEGs in the Cp2‐P/Cp2 group were significantly enriched in ‘transport’, ‘localisation’ and ‘transporter activity’ (Figure 4A). In addition, in mice, the downregulated DEGs of strain Cp2 were predominantly enriched in the category of ‘generation of precursor metabolites and energy’ (Figure 5A), and no upregulated DEGs were enriched. For comparisons between the two hosts, we found that most upregulated bacterial DEGs were enriched in the category of biological process, including ‘cellular nitrogen compound biosynthetic’, ‘organonitrogen compound metabolic’, ‘organonitrogen compound biosynthetic’ and ‘oxidation–reduction processes’ (Figure 5B). In the cellular component, the upregulated bacterial DEGs were enriched in the same types of pathways as those in the Cp2‐P/Cp2 group, with only differences in number. Furthermore, in the molecular function category, ‘structural constituent of ribosome’ and ‘structural molecule activity’ were present in the upregulated bacterial DEGs of Cp2‐P/Cp2‐A group (Figure 5B). These results indicated that strain Cp2 caused upregulation of DEGs after infection of alfalfa and mice, and the enriched pathways of these upregulated bacterial DEGs were mostly concentrated in the biological process. Moreover, the types of these upregulated bacterial DEGs in the cellular component category in mice were the same as those enriched by strain Cp2 when infecting alfalfa.

**FIGURE 6:**
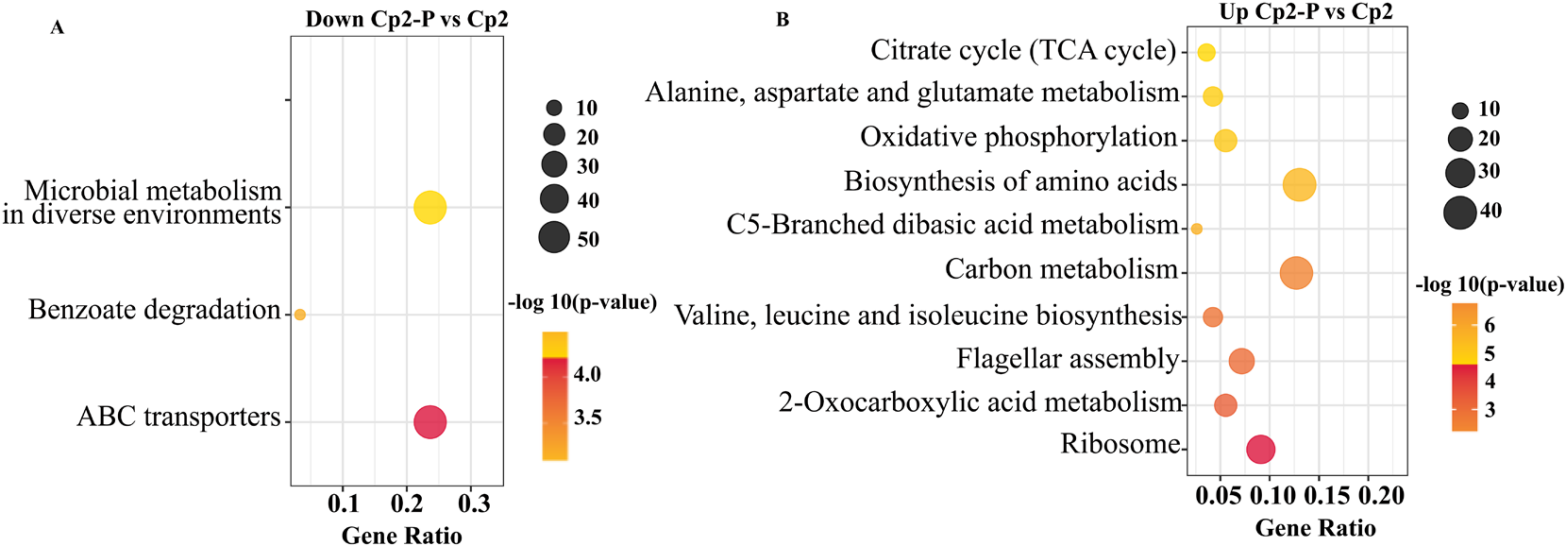
The KEGG pathways containing the DEGs. (A) The KEGG annotated entries containing downregulated DEGs in the Cp2‐P/Cp2 group. (B) The KEGG annotated entries containing upregulated DEGs in the Cp2‐P/Cp2 group.

**FIGURE 7:**
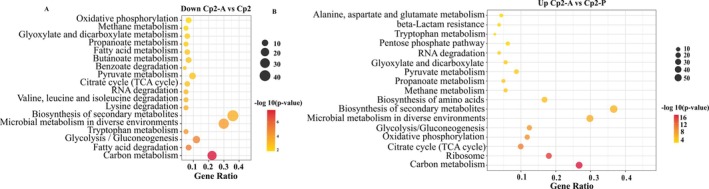
(A) The KEGG annotated entries containing downregulated DEGs in the Cp2‐A/Cp2 group. (B) The KEGG annotated entries containing upregulated DEGs in the Cp2‐A/Cp2‐P group.

To elucidate the functional roles of bacterial DEGs, we performed KEGG pathway enrichment analysis (Figure 6). In the Cp2‐P/Cp2 group, upregulated bacterial DEGs were significantly enriched in pathways such as ‘biosynthesis of amino acids’, ‘carbon metabolism’ and ‘ribosome’ (Figure 6A). Conversely, the downregulated bacterial DEGs were enriched in ‘ABC transporters’, ‘microbial metabolism in diverse environments’ and ‘benzoate degradation’ (Figure 6B). Notably, unlike the GO pathway analysis, no upregulated bacterial DEGs were enriched in any pathway. In the Cp2‐A/Cp2 group, the most downregulated bacterial DEGs were related to the ‘biosynthesis of secondary metabolites’, ‘microbial metabolism in diverse environments’ and ‘carbon metabolism’ (Figure 7A). Similarly, we also compared the two hosts. The results found that the most enriched upregulated bacterial DEGs were present in ‘biosynthesis of secondary metabolites’, ‘microbial metabolism in diverse environments’, ‘carbon metabolism’ and ‘ribosome’ for the Cp2‐A/Cp2‐P group (Figure 7B). These results showed that most of the upregulated bacterial DEGs were enriched in metabolic and ribosomal pathways after strain Cp2 infected either alfalfa or mice.

### 3.4 Toxicity Factor Analysis of DEGs

**FIGURE 8:**
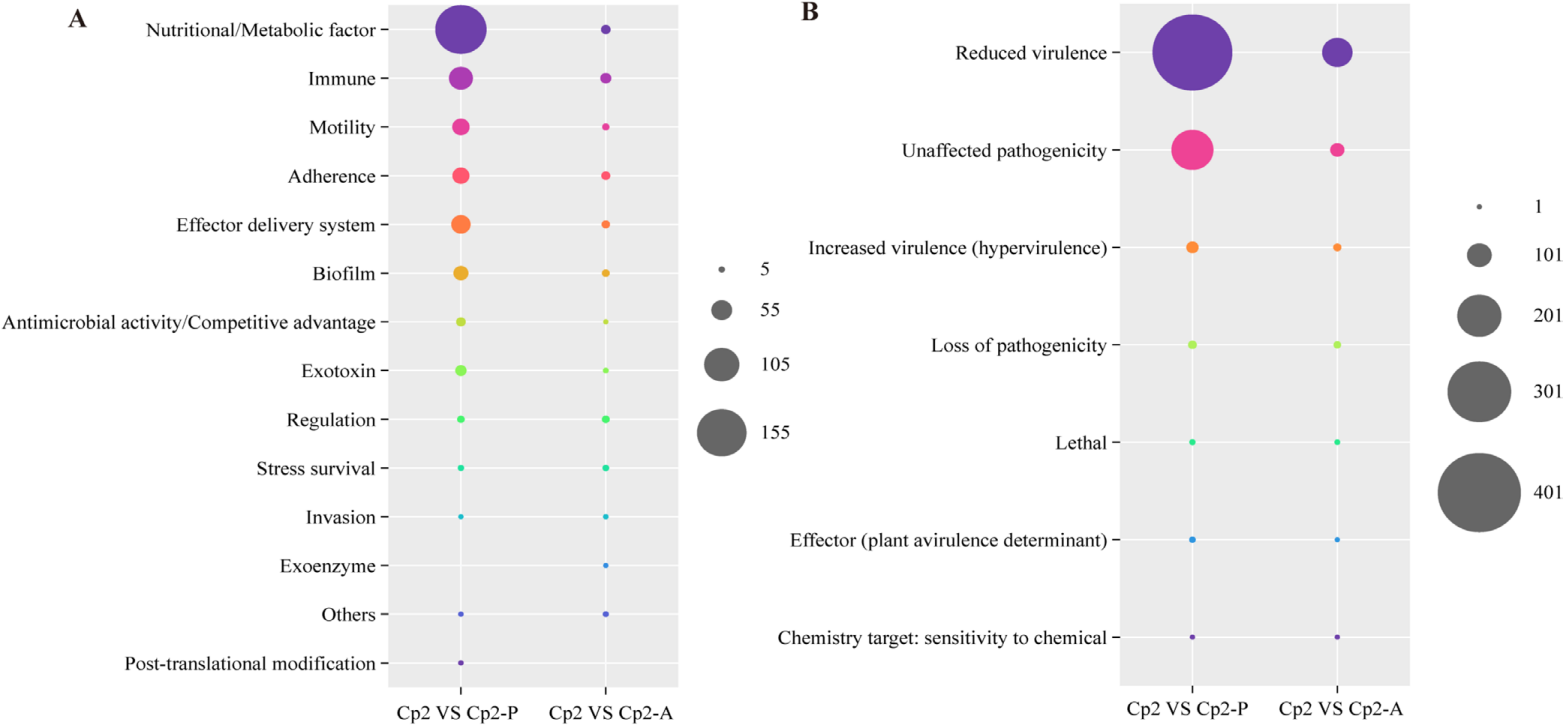
Toxicity factor analysis and pathogen‐host interactions analysis of DEGs. (A) The 14 pathways of virulence factors encoded by DEGs. (B) The seven pathways of pathogenic genes matched by DEGs in PHI‐base.

All bacterial DEGs were analysed against the VDFB (Virulence Factors of Bacterial Pathogens) database (https://www.mgc.ac.cn/VFs/), revealing that the number of virulence factors annotated by the DEGs of strain Cp2 in alfalfa and mice was 454 (32.38%) and 108 (27.76%), respectively, with 34 shared between the two. In alfalfa, the DEGs of Cp2 were primarily enriched in virulence factors related to ‘nutritional‐metabolic factor’, ‘immune’, ‘motility’, ‘adherence’, ‘effector delivery system’ and ‘biofilm’ (Figure 8A). In mice, the virulence factors enriched by DEGs are predominantly related to the following pathways: ‘immune’, ‘adherence’, ‘effector delivery system’ and ‘biofilm’ (Figure 8A).

**FIGURE 9:**
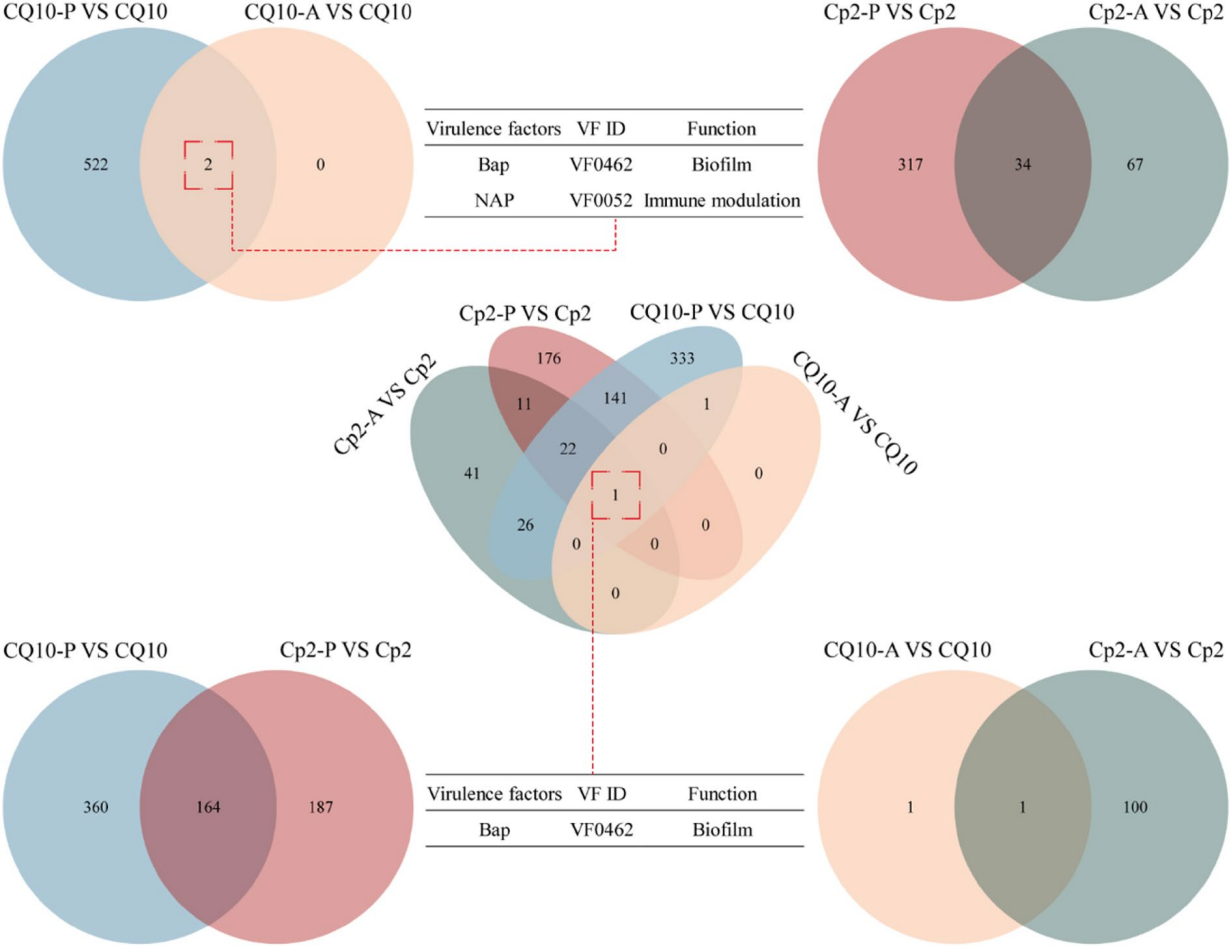
Venn analysis of virulence factors encoded by DEGs. CQ10: CQ10 bacterial cells. CQ10‐A: Animal samples inoculated with CQ10. CQ10‐P: Plant samples inoculated with CQ10; CQ10‐P versus CQ10: Virulence factors encoded by DEGs in CQ10‐P and CQ10. CQ10‐A versus CQ10: Virulence factors encoded by DEGs in CQ10‐A and CQ10. The same as below.

To further elucidate the cross‐kingdom virulence genes of Cp2, we compared the virulence factors encoded by bacterial DEGs in both alfalfa and mice using *P. alfalfa* CQ10 as a reference (Figure 9). The results indicate that strain Cp2 expressed a total of 34 virulence genes in both alfalfa and mice, while strain CQ10 consistently expressed 2 virulence genes, namely, biofilm‐associated protein *Bap* and immune modulation *NAP*. In alfalfa, the two strains co‐expressed 164 virulence genes, predominantly ‘nutritional‐metabolic factor (32.93%)’, ‘motility (13.41%)’ and ‘immune (12.80%)’. In mice, only one virulence gene was co‐expressed by the two strains: *Bap*. A Venn diagram analysis further revealed that among the virulence factors encoded by bacterial DEGs in the four groups, only *Bap* exhibited co‐expression (Figure 9). These findings indicate that *Bap* plays a significant role in the process of Cp2 infection in both alfalfa and mice.

### 3.5 Analysis of DEGs in Pathogenic Bacteria–Host Interactions

**FIGURE 10:**
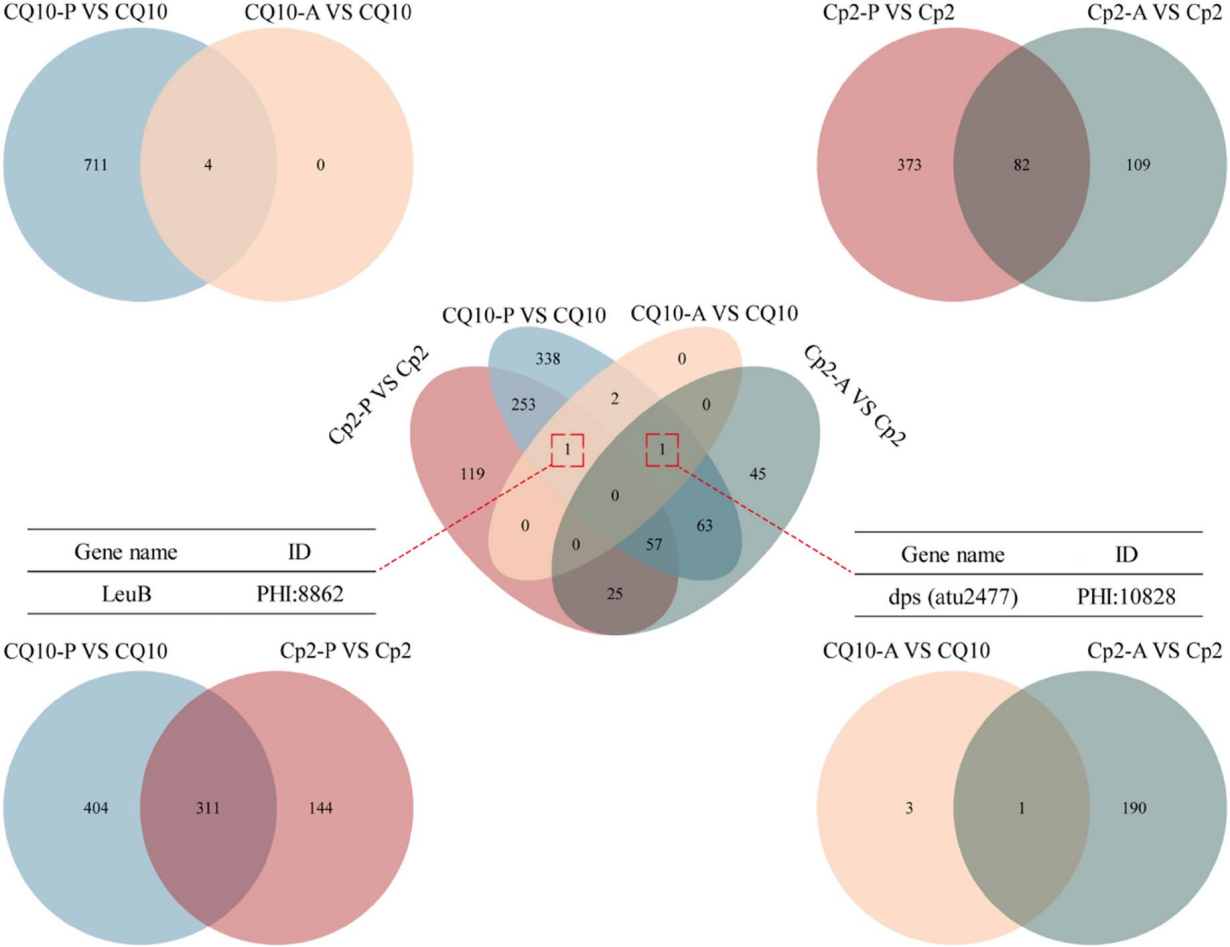
Venn analysis of pathogenic genes matched by DEGs in PHI‐base.

To identify bacterial DEGs related to pathogenesis‐related genes and functions, we annotated and analysed the DEGs using the pathogen–host interaction (PHI)‐base database (http://www.phi‐base.org/) (Figure 10). The results of functional annotation show that, out of 1969 bacterial DEGs, 82 genes were related to pathogenesis, and could be categorised into reduced virulence, unaffected pathogenicity and increased virulence (hypervirulence) (Figure 8B). Here, strain CQ10 was chosen for comparison. The PHI‐base enrichment analysis revealed that the two strains had 311 pathogenic genes when infecting alfalfa, but only one when infecting mice (Figure 10). A Venn diagram analysis revealed that *LeuB* was a pathogenic gene co‐expressed by both Cp2 and CQ10 in alfalfa, while *dpS* was co‐expressed in mice (Figure 10).

## 4 Discussion

### 4.1 Plant Pathogens Can Cause Cross‐Kingdom Diseases in Animals

Unlike the intraperitoneal injection mouse model established by Mohamaden et al. (2019), the pathogenicity of *E. persicina* Cp2 was investigated here using a gavage model in mice. After gavage, bacterial suspension‐treated mice developed severe lesions in the stomach, liver, spleen and kidney, as well as less severe pathological changes in the heart and lung tissues. In the Mohamaden et al. (2019) study, common symptoms included inflammation in lung tissues and necrosis in individual hepatocytes. In our study, the differential symptoms were that some of the gastric mucosal cells in the stomach were damaged. We found that strain Cp2 may adhere to and colonise the surface epithelial cells of the gastric mucosa after bacteria enter the stomach, potentially leading to further inflammatory reactions. The ongoing inflammatory response resulted in damage to the gastric mucosa. Combined with the findings from the Forage Pathology Laboratory of Gansu Agricultural University (Yao et al. 2022), Cp2 is identified as a plant and animal cross‐kingdom pathogenic bacterium. Based on observations of pathological tissue slices, Cp2 primarily induced pathological changes in the glomerular and tubular epithelium of mouse kidneys. Cp2 was also isolated from kidney tissue, further indicating that Cp2 can enter the kidneys of mice and cause harm. Additionally, the liver and spleen tissues exhibited moderate lesions. This may be attributed to the inflammatory response induced by the Cp2 or its secondary metabolites infiltrating these tissues after gavage. *E. persicina* produced biofilms that consist of exopolysaccharide (EPS) and 6.7% protein, and these components are completely secreted into the environment as slime (Kiessling et al. 2005). Biofilms are ubiquitous under natural conditions and are a major factor in human bacterial infections (Hall‐Stoodley et al. 2004). EPS in the external chemical structure may also induce inflammation and stimulate the infiltration of neutrophils and lymphocytes, as previously mentioned by Shin et al. (2016).

In this experiment, pathological symptoms showed that Cp2 was pathogenic in mice, and its virulence differed significantly from that of other cross‐kingdom pathogenic bacteria. For instance, *P. aeruginosa* , the bacterium that causes leaf spot disease in tobacco, was inoculated into mice via intraperitoneal injection, resulting in successful infection (Elrod and Braun 1942, 1941). Some mice injected with the 0.05 mL bacterial suspension died within 12 h. Similarly, Muraschi et al. (1965) reported mouse mortality within 24 h after intraperitoneal injection of a 10^9^ CFU/mL *Erwinia* suspension. However, in the study, mice did not die after inoculation with a 10^9^ CFU/mL Cp2 suspension. One potential reason may be that the pathogenicity of Cp2 to animals is weaker than that of other cross‐kingdom bacteria, and the robust immune mechanism of mice may have mitigated the infection. However, this needs further verification in later experiments. Another reason could be the differing administration methods, which lead to different circulation pathways of drugs. The administration method used in this experiment, the gavage method, more closely mimics the feeding behaviour of livestock compared to intraperitoneal injection (Henderson et al. 2002).

### 4.2 Virulence Genes Associated With the Plant and Animal Cross‐Kingdom Pathogenic Bacteria

The pathogenicity of pathogenic bacteria is primarily associated with the virulence factors they encode and produce. These virulence factors facilitate bacterial adherence to and invasion of host cells, leading to infection (Krausova et al. 2021; Sharma and Kanwar 2017; Rüger et al. 2022). Advanced transcriptomic profiling techniques have been employed in several studies to analyse gene expression during bacterial infection, emerging as a crucial tool for elucidating bacterial pathogenic mechanisms (Westermann et al. 2016). Specific genes involved in the autoinduction/transcriptional regulation of *quorum sensing* systems (bacterial cells produce and sense small signalling molecules to communicate with one another through a process termed quorum sensing; Whiteley et al. 2017) have been identified in animal and plant pathogens affecting *Erwinia* sp., including *expI*/*expR* and *carI*/*carR* found in *E. carotovora* and *expI*/*expR* in *E. chrysanthemi* (Cao et al. 2001). This study utilised dual RNA‐seq technology to determine that Cp2 can infect both alfalfa and mice, demonstrating its cross‐kingdom pathogenic potential. However, the screened DEGs were inconsistent with previous work. A total of 22 bacterial DEGs were identified from alfalfa and mice inoculated with strain Cp2, which were primarily enriched in the ‘ribosome’ GO and KEGG pathways. Ribosomes are typically regarded as constitutively functional protein synthesis centres not involved in regulatory processes (DeLivron and Robinson 2008). However, recent studies have shown that pathogenic bacteria can directly or indirectly regulate gene expression and virulence through changes in ribosome composition. For example, the Enteropathogenic *Escherichia coli* (EPEC) can regulate the components of the type III secretion system (T3SS) through it (Aroeti et al. 2025; Grant et al. 2003). Therefore, it is hypothesised that the ribosome pathway encoded by the DEGs of Cp2 may play a role in the virulence expression and regulation. However, the functional genes involved in the pathway need further functional verification and analysis.

When analysing the virulence factors encoded by bacterial DEGs, it was found that DEGs were primarily enriched in pathways such as ‘immunity’, ‘motility’ and ‘biofilm’ when Cp2 infected alfalfa and mouse hosts, which is similar to the virulence factors that other cross‐kingdom pathogenic bacteria depend upon (Hauser 2009; Pakbin et al. 2021; Indikova et al. 2014). This further illustrates that Cp2 relies on diverse virulence factors during host infection. Additionally, another plant and animal cross‐kingdom pathogenic bacterium, *P. agglomerans* CQ10, was selected for comparison. It was found that the only virulence factor co‐expressed by the two strains in alfalfa and mice was ‘Biofilm‐associated protein’ (*Bap*). This demonstrated that *Bap* plays a critical role in the pathogenic process of cross‐kingdom pathogenic bacteria. In vitro studies have shown that *Bap* contributes to the formation of pathogenic bacterial biofilms and the colonisation and adhesion of eukaryotic host cells (Lasa and Penadés 2006; Taglialegna et al. 2016). After the gene encoding the virulence factor is knocked out, the biofilm formation ability of bacteria is lost, and compensatory strains promote biofilm production (Cucarella et al. 2002; Heilmann et al. 1997; Muraschi et al. 1965). For example, all *Streptococcus aureus* strains expressing the *Bap* gene are strong biofilm producers, and the presence of *Bap* significantly increases the ability of *S. aureus* to colonise and persist in the breast in vivo (Cucarella et al. 2004). Additionally, many surface proteins similar to *Bap* have recently been shown to be involved in biofilm formation, host colonisation and adhesion in various bacterial species, such as *Burkholderia cepacian* (Huber et al. 2002), *P. fluorescens* (Hinsa et al. 2003) and *Enterococcus faecalis* (Toledo‐Arana et al. 2001). After aligning bacterial DEGs with the PHI‐base database, 82 pathogenic genes were identified to be commonly present in both alfalfa and mice. Similarly, when compared with CQ10, *leuB* was the pathogenic gene co‐expressed in inoculated alfalfa by the two strains, and *dps* was co‐expressed in inoculated mice. However, no co‐expression was identified for pathogenic genes during the interaction between the two strains and their respective hosts. This study has demonstrated that *leuB* is associated with the ability of pathogenic bacteria to synthesise leucine. The absence of *leuB* can lead to a significant decrease in the motility and biofilm formation ability of pathogens, reducing their ability to infect the host (Ren et al. 2014). *Dps* (atu2477) primarily contributes to the regulation of iron homeostasis in pathogenic bacteria (Yang et al. 2020). Moreover, iron ion binding was also enriched in the GO pathway. In summary, *Bap* is the key virulence gene in *E. persicina* Cp2 for both plant and animal infections, as well as in other cross‐kingdom pathogenic bacteria. In the future, we will focus on verifying its specific functionality.

## 5 Conclusions

This study established an alfalfa root infection and a mouse gavage model, demonstrating the pathogenicity of *E. persicina* Cp2 in both alfalfa and mice. Transcriptome analysis comprehensively revealed the cross‐kingdom virulence factors in Cp2, confirming its pathogenicity in two hosts. DEGs were primarily enriched in pathways related to ‘biofilm formation’, ‘immune evasion’, and ‘motility’, which are processes commonly associated with cross‐kingdom virulence. Comparative analysis with another cross‐kingdom pathogen, CQ10, highlighted the key role of *Bap* in mediating cross‐kingdom pathogenicity. The conserved involvement of *Bap* in biofilm formation and host colonisation across bacterial species further underscores its importance in cross‐kingdom infections. Notably, *leuB* and *dps* were identified as co‐expressed virulence genes during both alfalfa and mouse infections, revealing specific genetic determinants of cross‐kingdom pathogenicity of Cp2.

## Author Contributions

**Rong Huang:** writing – original draft, visualization, methodology. **Zhen‐Fen Zhang:** writing – review and editing, funding acquisition. **Tom Hsiang:** writing – review and editing. **Xiao‐Ni Liu:** writing – review and editing.

## Funding

This work was supported by Longyuan Young Talents Project of Gansu Province (LYYC‐2025‐03), the Fuxi Distinguished Talent Cultivation Project of Gansu Agricultural University (Gaufx‐05j01), the Science and Technology Support Project of the Gansu Forestry and Grassland Administration—Gansu Grassland Ecological Restoration and Management Project (LCJ20240172), the Department of Education Young Doctoral Researchers ‘In‐Enterprise & In‐Park’ Collaboration Project (2025QB‐051) and the National Natural Science Foundation of China (32060396).

## Conflicts of Interest

The authors declare no conflicts of interest.

## Supporting information


**Table S1:** Sequence of RT‐qPCR primers.
**Figure S1:** RTq‐PCR verification of DEGs.

## Data Availability

The data that support the findings of this study are available on request from the corresponding author. The data are not publicly available due to privacy or ethical restrictions.

## References

[emi470322-bib-0001] Aroeti, L. , N. Elbaz , R. Faigenbaum‐Romm , et al. 2025. “Formation of a Membraneless Compartment Regulates Bacterial Virulence.” Nature Communications 16: 3834. 10.1038/s41467-025-58829-9. PMC12019536 40268935

[emi470322-bib-0002] Brenner, D. J. , N. R. Krieg , and J. T. Staley . 2005. Bergey's Manual of Systematic Bacteriology (Second Edition, Volume 2 Part B, C). Springer US.

[emi470322-bib-0004] Büttner, D. , and U. Bonas . 2003. “Common Infection Strategies of Plant and Animal Pathogenic Bacteria.” Current Opinion in Plant Biology 6: 312–319. 10.1016/s1369-5266(03)00064-5. 12873524

[emi470322-bib-0005] Cao, H. , R. L. Baldini , and L. G. Rahme . 2001. “Common Mechanisms for Pathogens of Plants and Animals.” Annual Review of Phytopathology 39: 259–284. 10.1146/annurev.phyto.39.1.259. 11701866

[emi470322-bib-0007] Cucarella, C. , M. A. Tormo , E. Knecht , et al. 2002. “Expression of the Biofilm‐Associated Protein Interferes With Host Protein Receptors of *Staphylococcus aureus* and Alters the Infective Process.” Infection and Immunity 70: 3180–3186. 10.1128/IAI.70.6.3180-3186.2002. 12011013 PMC127991

[emi470322-bib-0008] Cucarella, C. , M. Á. Tormo , C. Úbeda , et al. 2004. “Role of Biofilm‐Associated Protein Bap in the Pathogenesis of Bovine *Staphylococcus aureus* .” Infection and Immunity 72: 2177–2185. 10.1128/IAI.72.4.2177-2185.2004. 15039341 PMC375157

[emi470322-bib-0009] DeLivron, M. A. , and V. L. Robinson . 2008. “ *Salmonella enterica* Serovar *Typhimurium* BipA Exhibits Two Distinct Ribosome Binding Modes.” Journal of Bacteriology 190: 5944–5952. 10.1128/JB.00763-08. 18621905 PMC2519513

[emi470322-bib-0010] Elrod, R. P. , and A. C. Braun . 1941. “A Phytopathogenic Bacterium Fatal to Laboratory Animals.” Sciences 94: 520–521. 10.1126/science.94.2448.520. 17809186

[emi470322-bib-0011] Elrod, R. P. , and A. C. Braun . 1942. “ *Pseudomonas aeruginosa*: Its Role as a Plant Pathogen.” Journal of Bacteriology 44: 633–645. 10.1128/jb.44.6.633-645.1942. 16560603 PMC374797

[emi470322-bib-0012] Gálvez, L. , J. Gil‐Serna , M. García‐Diaz , and D. Palmero . 2015. “First Report of a Garlic Bulb Rot Caused by *Erwinia persicina* in Europe.” Plant Disease 99: 723. 10.1094/PDIS-11-14-1195-PDN.

[emi470322-bib-0013] González, A. J. , J. C. Tello , and M. de Cara . 2005. “First Report of *Erwinia persicina* From *Phaseolus vulgaris* in Spain.” Plant Disease 89: 109. 10.1094/PD-89-0109C. 30795305

[emi470322-bib-0014] González, A. J. , J. C. Tello , and M. R. Rodicio . 2007. “ *Erwinia persicina* Causing Chlorosis and Necrotic Spots in Leaves and Tendrils of *Pisum sativum* in Southeastern Spain.” Plant Disease 91: 460. 10.1094/PDIS-91-4-0460A. 30781193

[emi470322-bib-0015] Grant, A. J. , M. Farris , P. Alefounder , P. H. Williams , M. J. Woodward , and C. D. O'Connor . 2003. “Co‐Ordination of Pathogenicity Island Expression by the *BipA* GTPase in Enteropathogenic *Escherichia coli* (EPEC).” Molecular Microbiology 48: 507–521. 10.1046/j.1365-2958.2003.t01-1-03447.x. 12675808

[emi470322-bib-0016] Hall‐Stoodley, L. , J. W. Costerton , and P. Stoodley . 2004. “Bacterial Biofilms: From the Natural Environment to Infectious Diseases.” Nature Reviews. Microbiology 2: 95–108. 10.1038/nrmicro821. 15040259

[emi470322-bib-0017] Hao, Y. , S. C. Winans , B. R. Glick , and T. C. Charles . 2020. “ *Erwinia persicina*: A Versatile Bacterium With Ecological Diversity.” Microbial Ecology 79: 345–357.

[emi470322-bib-0018] Hauser, A. R. 2009. “The Type III Secretion System of *Pseudomonas aeruginosa* : Infection by Injection.” Nature Reviews. Microbiology 7: 654–665. 10.1038/nrmicro2199. 19680249 PMC2766515

[emi470322-bib-0019] Heilmann, C. , M. Hussain , G. Peters , and F. Götz . 1997. “Evidence for Autolysin‐Mediated Primary Attachment of *Staphylococcus epidermidis* to a Polystyrene Surface.” Molecular Microbiology 24: 1013–1024. 10.1046/j.1365-2958.1997.4101774.x. 9220008

[emi470322-bib-0020] Henderson, S. O. , S. Swadron , and E. Newton . 2002. “Comparison of Intravenous Ketorolac and Meperidine in the Treatment of Biliary Colic.” Journal of Emergency Medicine 23: 237–241. 10.1016/s0736-4679(02)00524-3. 12426013

[emi470322-bib-0021] Hinsa, S. M. , M. Espinosa‐Urgel , J. L. Ramos , and G. A. O'Toole . 2003. “Transition From Reversible to Irreversible Attachment During Biofilm Formation by *Pseudomonas fluorescens* WCS365 Requires an ABC Transporter and a Large Secreted Protein.” Molecular Microbiology 49: 905–918. 10.1046/j.1365-2958.2003.03615.x. 12890017

[emi470322-bib-0023] Huber, B. , K. Riedel , M. Köthe , M. Givskov , S. Molin , and L. Eberl . 2002. “Genetic Analysis of Functions Involved in the Late Stages of Biofilm Development in *Burkholderia cepacia* H111.” Molecular Microbiology 46: 411–426. 10.1046/j.1365-2958.2002.03182.x. 12406218

[emi470322-bib-0024] Indikova, I. , M. Vronka , and M. P. Szostak . 2014. “First Identification of Proteins Involved in Motility of *Mycoplasma gallisepticum* .” Veterinary Research 45: 99. 10.1186/s13567-014-0099-2. 25323771 PMC4207318

[emi470322-bib-0026] Kiessling, P. , S. N. Senchenkova , M. Ramm , and Y. A. Knirel . 2005. “Structural Studies on the Exopolysaccharide From *Erwinia persicina* .” Carbohydrate Research 340: 1761–1765. 10.1016/j.carres.2005.06.004. 15992784

[emi470322-bib-0027] Kim, J. , S. Lee , D. H. Park , and C. S. Oh . 2022. “Isolation of *Erwinia persicina* From Rock Surfaces and Its Potential Role in Mineral Weathering.” Applied and Environmental Microbiology 88: e00345‐22. 10.1016/j.ecoleng.2017.01.023.

[emi470322-bib-0028] Kishitani, S. , T. Takanami , M. Suzuki , et al. 2000. “Compatibility of Glycinebetaine in Rice Plants: Evaluation Using Transgenic Rice Plants With a Gene for Peroxisomal Betaine Aldehyde Dehydrogenase From Barley.” Plant, Cell & Environment 23: 107–114. 10.1046/j.1365-3040.2000.00527.x.

[emi470322-bib-0029] Krausova, G. , I. Hynstova , R. Svejsti , I. Mrvikova , and R. Kadlec . 2021. “Identification of Synbiotics Conducive to Probiotics Adherence to Intestinal Mucosa Using an In Vitro Caco‐2 and HT29‐MTX Cell Model.” Processes 9: 569. 10.3390/pr9040569.

[emi470322-bib-0030] Lasa, I. , and J. R. Penadés . 2006. “Bap: A Family of Surface Proteins Involved in Biofilm Formation.” Research in Microbiology 157: 99–107. 10.1016/j.resmic.2005.11.003. 16427771

[emi470322-bib-0063] Lee, H. B. , J. P. Hong , and S. B. Kim . 2010. “First Report of Leaf Blight Caused by *Pantoea agglomerans* on Rice in Korea.” Plant Disease 94: 1372. 10.1094/PDIS-05-10-0374. 30743637

[emi470322-bib-0031] Livak, K. J. , and T. D. Schmittgen . 2001. “Analysis of Relative Gene Expression Data Using Real‐Time Quantitative PCR and the 2^−ΔΔCt^ Method.” Methods 25: 402–408. 10.1006/meth.2001.1262. 11846609

[emi470322-bib-0032] Lum, K. K. , and I. M. Cristea . 2016. “Proteomic Approaches to Uncovering Virus‐Host Protein Interactions During the Progression of Viral Infection.” Expert Review of Proteomics 13: 325–340. 10.1586/14789450.2016.1147353. 26817613 PMC4919574

[emi470322-bib-0034] Mohamaden, W. I. , Z. F. Zhang , I. M. Hegab , and S. L. Shi . 2019. “Experimental Infection in Mice With *Erwinia persicina* .” Microbial Pathogenesis 130: 38–43. 10.1016/j.micpath.2019.01.050. 30826431

[emi470322-bib-0035] Muraschi, T. F. , M. Friend , and D. Bolles . 1965. “ *Erwinia*‐Like Microorganisms Isolated From Animal and Human Hosts.” Applied Microbiology 13: 128–131. 10.1128/am.13.2.128-131.1965. 14325868 PMC1058210

[emi470322-bib-0036] Nechwatal, J. , and S. Theil . 2019. “ *Erwinia persicina* Associated With a Pink Rot of Parsley Root in Germany.” Journal of Plant Diseases and Protection 126: 161–167. 10.1007/s41348-018-0200-6.

[emi470322-bib-0037] O'Hara, C. M. , A. G. Steigerwalt , B. C. Hill , J. M. Miller , and D. J. Brenner . 1998. “First Report of a Human Isolate of *Erwinia persicinus* .” Journal of Clinical Microbiology 36: 248–250. 10.1128/JCM.36.1.248-250.1998. 9431957 PMC124844

[emi470322-bib-0038] Orel, D. C. 2020. “ *Erwinia persicina* as the New Causal Agent of Lettuce Soft Rot.” European Journal of Plant Pathology 158: 223–235. 10.1007/s10658-020-02068-9.

[emi470322-bib-0040] Pakbin, B. , W. M. Brück , and J. W. A. Rossen . 2021. “Virulence Factors of Enteric Pathogenic *Escherichia coli*: A Review.” International Journal of Molecular Sciences 22: 9922. 10.3390/ijms22189922. 34576083 PMC8468683

[emi470322-bib-0041] Rahme, L. G. , E. J. Stevens , S. F. Wolfort , J. Shao , R. G. Tompkins , and F. M. Ausubel . 1995. “Common Virulence Factors for Bacterial Pathogenicity in Plants and Animals.” Science 268: 1899–1902. 10.1126/science.7604262. 7604262

[emi470322-bib-0042] Ren, Z. G. , W. J. Jiang , X. Y. Ni , et al. 2014. “Multiplication of *Acidovorax citrulli* in Planta During Infection of Melon Seedlings Requires the Ability to Synthesize Leucine.” Plant Pathology 63: 784–791. 10.1111/ppa.12156.

[emi470322-bib-0043] Rüger, N. , M. P. Szostak , and S. Rautenschlein . 2022. “The Expression of *GapA* and *CrmA* Correlates With the *Mycoplasma gallisepticum* In Vitro Infection Process in Chicken TOCs.” Veterinary Research 53: 66. 10.1186/s13567-022-01085-2. 36056451 PMC9440553

[emi470322-bib-0046] Sharma, S. , and S. S. Kanwar . 2017. “Adherence Potential of Indigenous Lactic Acid Bacterial Isolates Obtained From Fermented Foods of Western Himalayas to Intestinal Epithelial Caco‐2 and HT‐29 Cell Lines.” Journal of Food Science and Technology 54: 3504–3511. 10.1007/s13197-017-2807-1. 29051645 PMC5629159

[emi470322-bib-0047] Shin, J. S. , J. Y. Jung , S. G. Lee , et al. 2016. “Exopolysaccharide Fraction From *Pediococcus pentosaceus* KFT18 Induces Immunostimulatory Activity in Macrophages and Immunosuppressed Mice.” Journal of Applied Microbiology 120: 1390–1402. 10.1111/jam.13099. 26895351

[emi470322-bib-0049] Su, J. , B. Yao , R. Huang , X. Liu , Z. Zhang , and Y. Zhang . 2024. “Cross‐Kingdom Pathogenesis of *Pantoea alfalfa* CQ10: Insights From Transcriptome and Proteome Analyses.” Microorganisms 12: 2197. 10.3390/microorganisms12112197. 39597586 PMC11596184

[emi470322-bib-0050] Taglialegna, A. , S. Navarro , S. Ventura , et al. 2016. “Staphylococcal Bap Proteins Build Amyloid Scaffold Biofilm Matrices in Response to Environmental Signals.” PLoS Pathogens 12: e1005711. 10.1371/journal.ppat.1005711. 27327765 PMC4915627

[emi470322-bib-0051] Toledo‐Arana, A. , J. Valle , C. Solano , et al. 2001. “The Enterococcal Surface Protein, Esp, Is Involved in *Enterococcus faecalis* Biofilm Formation.” Applied and Environmental Microbiology 67: 4538–4545. 10.1128/AEM.67.10.4538-4545.2001. 11571153 PMC93200

[emi470322-bib-0052] Wasendorf, C. , S. Schmitz‐Esser , C. J. Eischeid , et al. 2022. “Genome Analysis of *Erwinia persicina* Reveals Implications for Soft Rot Pathogenicity in Plants.” Frontiers in Microbiology 13: 1001139. 10.3389/fmicb.2022.1001139. 36386708 PMC9650351

[emi470322-bib-0053] Westermann, A. J. , K. U. Förstner , F. Amman , et al. 2016. “Dual RNA‐Seq Unveils Noncoding RNA Functions in Host‐Pathogen Interactions.” Nature 529: 496–501. 10.1038/nature16547. 26789254

[emi470322-bib-0054] Whiteley, M. , S. P. Diggle , and E. P. Greenberg . 2017. “Progress in and Promise of Bacterial Quorum Sensing Research.” Nature 551: 313–320. 10.1038/nature24624. 29144467 PMC5870893

[emi470322-bib-0056] Yan, J. J. , Z. Y. Lin , R. Q. Wang , et al. 2018. “First Report of *Erwinia persicina* Causing Pink Disease in *Flammulina velutipes* (Enoki Mushroom) in China.” Plant Disease 103: 1014. 10.1094/PDIS-06-18-0950-PDN.

[emi470322-bib-0057] Yang, J. , X. Pan , Y. Xu , et al. 2020. “ *Agrobacterium tumefaciens* Ferritins Play an Important Role in Full Virulence Through Regulating Iron Homeostasis and Oxidative Stress Survival.” Molecular Plant Pathology 21: 1167–1178. 10.1111/mpp.12969. 32678502 PMC7411545

[emi470322-bib-0058] Yao, B. , R. Huang , Z. F. Zhang , and S. L. Shi . 2022. “Seed‐Borne *Erwinia persicina* Affects the Growth and Physiology of Alfalfa (*Medicago sativa* L.).” Frontiers in Microbiology 13: 891188. 10.3389/fmicb.2022.891188. 35694312 PMC9178255

[emi470322-bib-0059] Zhang, Y. J. , X. N. Liu , X. Y. Li , et al. 2022. “Physicochemical Properties and Antibiosis Activity of the Pink Pigment of *Erwinia persicina* Cp2.” Agriculture 12: 1641. 10.3390/agriculture12101641.

[emi470322-bib-0060] Zhang, Z. F. 2013. Seed‐Borne Bacteria of Lucerne (Medicago sativa) and Their Pathogenicity. Lanzhou University.

[emi470322-bib-0062] Zhang, Z. F. , and Z. B. Nan . 2012. “First Report of *Erwinia persicinus* Causing Wilting of *Medicago sativa* Sprouts in China.” Plant Disease 96: 454. 10.1094/PDIS-10-11-0909. 30727103

[emi470322-bib-0061] Zhang, Z. F. , and Z. B. Nan . 2014. “ *Erwinia persicina*, a New Necrosis and Wilt Threat for Grain Legumes and Forage Legumes Production in Agriculture.” European Journal of Plant Pathology 139: 343–352. 10.1007/s10658-014-0390-0.

