# Supplementary material for: Transcriptome Analysis Reveals Cross‐Kingdom Virulence Factors in Erwinia persicina Cp2
Source: Environ Microbiol Rep. 2026 Mar 11;18(2):e70322. doi: 10.1111/1758-2229.70322 (PMC13140447; doi:10.1111/1758-2229.70322)
Supplement: Supplementary file 1 — Table S1: Sequence of RT‐qPCR primers. Figure S1: RTq‐PCR verification of DEGs. [file EMI4-18-e70322-s001.docx]

**Table S1** Sequence of RT-qPCR primers.

| Gene | Forward primer（5`-3`） | Reverse primer（5`-3`） |
| --- | --- | --- |
| K6R05_RS11135 | CGCTGGAAGATGTGCTGGTGAC | GCATAAGTTACGCCCAGGGTGTC |
| K6R05_RS11145 | CGACGACCGCAAATTCCTTGAAATC | GGCGAGAAGTCATCCATGCTGTC |
| K6R05_RS18735 | GCAGCAGGTGAGAAGAGTGATTGAC | TTCGTGGTGACTTTGAGCAGATCG |
| K6R05_RS17085 | TAGTACAGGTCGTGGCTGCTCATC | GCAATCATCCCGCCCAGAATACC |
| K6R05_RS04870 | CGACGGGATTGTGAAGCCTTAGG | CAGACACGCCACTAACAGACTAGC |
| K6R05_RS19660 | GTGAAGCTGGCAGTGCAGGAG | TTAGAAGGACGGCGACTAATCTTGC |
| K6R05_RS02175 | CGGATGTCAGCTTCGGTACTTTCG | CGGCTTCTCGGTGATCGGTTTG |
| K6R05_RS02160 | CCTGCACTTGCTGAAGAAGGTAGAG | CACCCAGTTTGTGACCAACCATTTC |
| K6L24_RS05045 | GCAAACGACGAAAACTACGCACTAG | AACAGACACGCCACTGACAAACTAG |
| K6L24_RS13355 | TGGGCGTTAAGCAGGAAGAAGTTG | ATCAATAGCAGCCTGAACGGTAGTG |
| K6L24_RS22220 | CCCAGTCTCGTGAAGTGTCTTTAGC | CGATACCGTCTTCCAGCAGCATC |
| K6L24_RS06145 | GCACGGCGGGCTGAAGATATTC | CCCTGATAACCACTGAGCAGCATTC |
| K6L24_RS00700 | TTTCAACGGACCTGGGTTTCTCTTC | GGCGATACTGGATACGGTAATCACG |
| K6L24_RS01060 | GCTCAACGACGGCACAAAGATTTC | TGGCTCAACAAGACCTTCAACACC |
| K6L24_RS09815 | AGTCACATTGCTAACGGCGATATGG | GATACTGGCGACAATACCGAAGACC |
| K6L24_RS16845 | ACCGAGTGGACATCCGAAGAGTAG | TCGCCTTCACACTTGCCTTCAC |
| *rnpB* | CTTCGACAGCATGGATGACTTCTC | GCACCTTACCAATCAGCTCTTCAG |


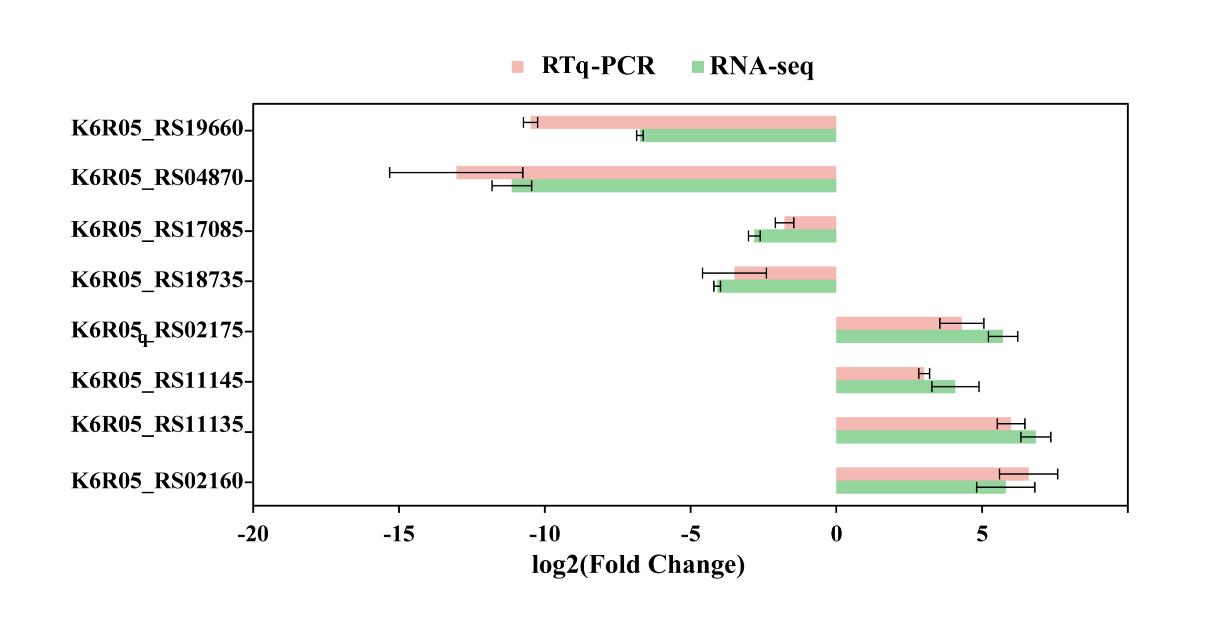


**FiguresS1** RTq-PCR verification of DEGs.
